# Attitude and perception towards vaccination against poliomyelitis in Peshawar, Pakistan

**DOI:** 10.11606/s1518-8787.2021055003478

**Published:** 2021-11-23

**Authors:** Shafique Farheen, Mahreen ul Hassan, Hina Nayab, Noreen Asim, Nazia Akbar, Nuzhat Shafi, Sadaf Manzoor, Freek van Eeden, Shaqaut Ali

**Affiliations:** I University of Sheffield Department of Biomedical Science Sheffield UK University of Sheffield. Department of Biomedical Science. Sheffield, UK; II Shaheed Benazir Bhutto Women University Department of Microbiology Peshawar Pakistan Shaheed Benazir Bhutto Women University. Department of Microbiology. Peshawar, Pakistan; III University of Sheffield Department of Molecular Biology and Biotechnology Sheffield UK University of Sheffield. Department of Molecular Biology and Biotechnology. Sheffield, UK; IV Sarhad University of Science and Information Technology Institute of Biological Sciences Peshawar Pakistan Sarhad University of Science and Information Technology. Institute of Biological Sciences. Peshawar 25000, Pakistan; V The University of Agriculture Institute of Biotechnology and Genetic Engineering Division of Genomics and Bioinformatics Peshawar Pakistan The University of Agriculture. Institute of Biotechnology and Genetic Engineering. Division of Genomics and Bioinformatics. Peshawar, Pakistan; VI University of Azad Jammu and Kashmir Department of Zoology Muzaffarabad Pakistan University of Azad Jammu and Kashmir. Department of Zoology. Muzaffarabad, Pakistan; VII Hazara University Mansehra Department of Biotechnology and Genetic Engineering Hazara Pakistan Hazara University Mansehra. Department of Biotechnology and Genetic Engineering. Hazara, Pakistan; VIII Islamia College University Department of Statistics Peshawar Khyber Pakhtunkha Pakistan Islamia College University. Department of Statistics. Peshawar, Khyber Pakhtunkha, Pakistan; IX Government College University Lahore Faculty of Science Department of Zoology Lahore Pakistan Government College University Lahore. Faculty of Science. Department of Zoology. Applied Entomology and Medical Toxicology Laboratory. Lahore, Pakistan

**Keywords:** Poliomyelitis, prevention & control, Poliovirus Vaccines, Vaccination Refusal, Vaccination Coverage, Health Knowledge, Attitudes, Practice

## Abstract

**OBJECTIVE:**

This research aimed to quantitatively assess the general public's awareness, attitude and perception of polio and its vaccination in Peshawar KPK, Pakistan.

**METHODS:**

We conducted a survey-based study to understand the surge in polio cases from 2015 to 2019 in the Peshawar city of the Khyber Pakhtunkhwa (KPK), Pakistan. A pre-tested questionnaire-based study was conducted in 2019 to assess the attitude and general perception of residents of Peshawar KPK towards polio vaccination.

**RESULTS:**

Out of 241 country-wide polio cases, 63 (26.1%) polio cases were reported in Peshawar city from 2015–2019. The questionnaire revealed that individuals between 18–30 years of age had sufficient knowledge (65.1%) about polio. Male and female participants had equal awareness (~ 43%). Participants with higher education (45.9%), those with better financial status (49.5%), individuals with children < 5 years of age (46.4%), and those who had experience of a polio patient (63.1%) had better knowledge. Participants inhabiting the central city were better aware (50.5%) of polio than individuals living in the outskirts.

**CONCLUSION:**

The data indicated that poor knowledge and negative attitudes of people towards polio vaccination are the main causes of the polio eradication program's failure. Moreover, religious beliefs, unchecked migration between the Pak-Afghan border, and lack of knowledge about polio vaccination are identified as critical barriers to polio eradication.

## INTRODUCTION

Earlier in the 20^th^ century, polio was an epidemic disease that caused paralysis in thousands of children^1^. The collaborative efforts of the Global Polio Eradication Initiative (GPEI), Centre for Disease Control and Prevention (CDC) and World Health Organization (WHO) with other parties successfully eradicated poliovirus using surveillance, awareness campaigns and massive immunisation in most of the world. However, two neighbouring countries are still considered endemic for the wild poliovirus type 1 (WPV1): Pakistan and Afghanistan^2^. The frequent migration of people between the countries in the Pakistan-Afghanistan border in Khyber Pakhtunkhwa (KPK) furthers the transmission of the virus. Disease control is necessary to restrict the virus's resurgence to a smaller area, i.e., Pakistan^3^.

Viruses remain infectious for extended periods and are resistant to decontamination processes used in drinking or wastewater treatment^4^. Therefore, insufficient knowledge about infectious agents and unhygienic conditions raise people's susceptibility to pandemics like cholera, COVID 19 and poliomyelitis^5^.

In contrast to the global trend, the number of polio cases has increased at an alarming rate in Pakistan. In 2014, out of 359 cases reported globally 306 were from Pakistan^2^. The most affected areas of Pakistan were KPK, Federally Administered Tribal Areas (FATA) and Baluchistan. Studies reveal that the upsurge of polio incidences throughout these years has been closely related to the lack of security of polio workers, and limited vaccine accessibility^6^.

The condition can be efficiently prevented by the administration of a vaccine in childhood. In Pakistan, clinical camps and door-to-door oral vaccination of every child under the age of five are implemented to ensure widespread availability of the vaccine^7^. Awareness campaigns, counselling exercises for parents in risk areas and media coverage have been planned and executed to make Pakistan polio free^8^.

Despite all the government's efforts to eradicate poliovirus from the country, the cases dramatically increased. A few religious and political leaders believe that the immunisation may cause infertility and Human Immuno-deficiency Virus (HIV) infection^9^. This misconception led to hostility towards healthcare workers even caused life-threatening situations. Moreover, the reported cases were from areas where literacy rate is very low^10^. Another important risk factor is insufficient sanitation, which remains an ignored aspect in disease prevention^11^. In addition to WPV1, the advent of Vaccine-derived Poliovirus (VDPV) from water sources could also intensify the incidence of the disease^12^. The global community is concerned about the presence of the poliovirus in Pakistan since it could cause a resurgence in polio-free countries. Until 2014, cases were identified from Peshawar KPK citizens^13^, but the disease's current status in the city is undetermined.

Our study aimed to investigate the socio-economic factors and epidemiology of poliomyelitis in Peshawar. It could help tackle the barriers responsible for the increased incidence of polio in the area.

## METHODS

### Study Location and Settings

A survey-based study was designed and conducted from March 2019 to September 2019 in the Peshawar city of Khyber Pakhtunkhwa (KPK), Pakistan. Peshawar is situated in Peshawar's broad valley near the eastern end of the historic Khyber Pass, close to the border with Afghanistan^14^. The area was selected due to the region being a “conveyer belt” for polio transmission as described by an Independent Monitoring Board of the Global Polio Eradication Initiative (GPEI)^6^.

This study complied with the STROBE guidelines for observational research^15^ and was conducted in view of the rise in polio incidence from 2015–2019 despite a very active Polio Eradication Initiative (PEI) program run by the government of Pakistan and funded by the World Health Organisation (WHO)^16^. From March to September 2019, the participants completed a pre-tested questionnaire at places like shopping malls, hospitals, and academic institutions. People from the city and the suburbs participated in our study, but individuals visiting or living in the area for a limited time were excluded. For the convenience of participants, a translated version of the questionnaire was used; however, if participants could not complete the questionnaire due to literacy problems, the interviewer-assisted technique was used for data collection.

The study also included responses from polio health-workers, which are the backbone of the polio eradication scheme and are directly affected by the negative attitude of people towards polio immunization.

### Data Sources

Data regarding polio cases from 2015–2019 obtained from the National surveillance Cell Federal EPI Office, administered by the National Institute of Health (NIH) in Islamabad, Pakistan, were confirmed by visiting the residences of the affected.

### Study Duration

The surveillance study duration was from March 2019 to September 2019.

The polio health workers were interviewed during the polio campaign held in April 2019.

### Sample Size and Eligibility Criteria

A questionnaire was designed to assess the local population's attitude towards polio and the vaccination program. For assessment, the sample size was calculated using Raosoft software with the power set to 80%, the response distribution at 50%, a confidence interval of 95%, and an error margin of 5% leading to 384 participants in Peshawar. For this study, all clinically healthy people aged 18 years and over were deemed eligible. The participants who were unable to take part in this study, refused to give any details, or were temporary residents were excluded.

Another questionnaire was designed to interview the polio health workers to assess the difficulties they face in the field during polio campaigns. The interview was structured to identify the possible barriers to polio vaccination.

### Sampling

A convenience non-probability sampling approach was used in this study. The method selected subjects due to their easy accessibility and proximity to the researchers.

For polio workers interviews, both male and female health workers and volunteers participating in the polio eradication campaign at the time of the study were approached at their workplaces and residences.

### Designing and Validating the Questionnaire

A questionnaire was designed after a detailed review of relevant literature. It was written in English, translated into Urdu for participants, and later translated back to English for analysis. The questionnaire was created with the guidance of two physicians, an expert in public health and infectious diseases, a pharmacist, and a sociologist. A pilot study was performed on twenty-five random individuals. By considering other published literature, the modifications suggested by the participants in the questionnaire were adopted before distribution among test subjects. The responses obtained from participants of the pilot study were excluded from the final data. For testing reliability, Cronbach's alpha test was applied. The reliability coefficient of the questionnaire was 0.74.

The questionnaire consisted of four sections with 45 questions. In section one, nine questions evaluated demographic data such as age, gender, ethnicity, income and literacy, etc. The second section was comprised of 15 questions to assess participants’ knowledge about poliovirus, signs and symptoms, and vaccination against the virus. Knowledge was measured by giving a score of 1 to the right answer and 0 to the wrong answer. The scale measured knowledge from a maximum score of 15 to a minimum of 0. Scores < 9 were taken as poor, ≥ 9 as sufficient knowledge of polio disease. In section three, the evaluation of participants was based on eight attitude-related statements. In the final segment, the participants’ perception of polio vaccination was assessed by their responses to 13 questions.

### Data Analysis

All the information gathered through this study was analysed by Microsoft Excel 16.0 and GraphPad version 9.0.0.

### Ethical Approval and Consent to Participate

This study was approved by the Research and Ethics Committee, University of Agriculture, Peshawar-Pakistan. A filled and signed informed consent form was collected from each participant before the participation. All the information of the participants is kept confidential.

## RESULTS

**Figure 1:**
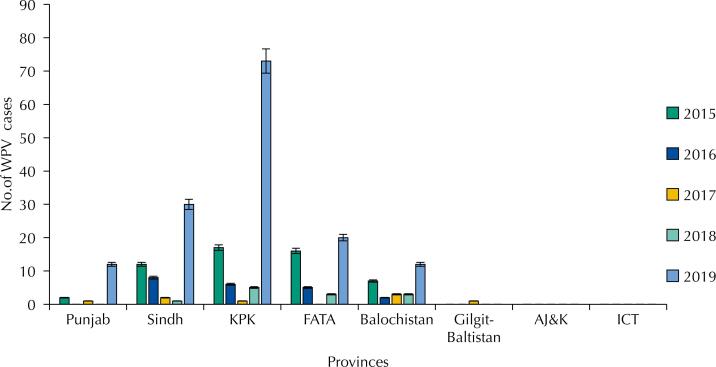
Province-wise WPV cases from 2015–2019 (NIH, Pakistan).

From 2015 to 2019, there were 241 confirmed WPV (including VDPV) cases reported across the country. About 54 (22.4%) cases were reported in 2015, 20 (8.29%) cases in 2016, 8 (3.3%) in 2017 and 12 (4.97%) in 2018 (Figure 1). Worryingly, approximately 147 (60.9%) cases were reported in 2019 alone. Over five years, 146 cases came from KPK and FATA, and only Sindh got close to them with 53 cases in the period^17^.

Table 1 shows that, during 2015–2019, 70 WPV cases were reported in Peshawar. Out of 70 suspected cases, 63 (90%) cases were confirmed WPV patients, and 7 (10%) claims were excluded from incomplete information or misdiagnosis. Among the confirmed cases, 31 (49.2%) cases had received insufficient doses of vaccine, and 32 (52.7 %) patients were not given any type of vaccine at all.

**Table 1:** Poliomyelitis cases in Peshawar (2015–2019) and the number of 3 WPV serotypes.

Year	No. of Patients	Sex		Mean age at disease onset (in months)	Partially vaccinated patients (%)	Serotypes (%)		
		Male (%)	Female (%)			1	2	3
2015	8	3 (37.5%)	5 (62.5 %)	20	5 (62.5 %)	8 (100%)	–	–
2016	9	4 (44.5%)	5 (55.5 %)	15	5 (55.5 %)	8 (88 %)	–	1 (12%)
2017	8	3 (37.5%)	5 (62.5 %)	07	4 (50 %)	8 (100%)	–	–
2018	28	12 (42.9%)	16 (57.1 %)	13.4	15 (53.5 %)	28 (100%)	–	–
2019	10	5 (50.0%)	5 (50.0 %)	12.1	2 (20.0 %)	10 (100%)	–	–

The data on polio positive patients from 2015–2019 was collected from the National surveillance Cell Islamabad. About 63 positive polio patients were enrolled in these five years from the Peshawar region of KPK province, Pakistan. Among these positive cases, 8 (12%) cases were reported in 2015, with 5 males and 3 females. In 2016, 9 (14.2%) children were diagnosed with WPV (5 males and 4 females), while in 2017, there were 8 (12.6%) new polio cases, including 5 males and 3 female patients. In 2018, despite local government efforts, polio cases increased with 10 (15.8 %) new cases (5 males and 5 females), spiking in 2019, when 28 (44.4%) cases, including 16 males and 12 females were reported in Peshawar city alone. The mean ages of the patients during the polio onset ranged between 12–20 months from 2015 to 2019 (Table 1). We observed that the incidence of poliomyelitis was slightly higher in the patients getting polio vaccine due to inadequate vaccine storage or mishandling, or most likely insufficient dosing (see below).

Table 2 shows the number of doses for partially vaccinated children analysed in children of different age groups, ranging from 1–12 months (m), 13–24 m, 24–36 m, and 37–48 m. Out of 31 cases, 10 children aged 1–12 m were partially immunised, and 22 were unvaccinated. In the group aged 13–24 months, 11 children were vaccinated, and 10 were unvaccinated, while in the group of 24–36 month-olds, all six children were vaccinated. All four children between 37–48 months old were vaccinated. About 60 children from all three age groups were randomly selected, and their parents were interviewed. The children who had received the complete polio vaccine course (9–12 doses) remained healthy and did not show any symptoms related to the disease or any side effect during or after the study.

**Table 2:** Distribution of polio cases; mean age (on disease onset) with vaccination and number of doses.

No. of doses/age in months	Partially vaccinated (n = 31)					Unvaccinated (n = 32)	Completely vaccinated (n = 60)
	1^st^ dose	2^nd^ dose	3^rd^ dose	More than 3 doses	Total no. of cases received vaccination	No. of cases received Zero doses	No. of cases received all doses
01–12 months	02	02	06	00	10	21	20
13–24 months	02	01	08	00	11	10	15
24–36 months	02	01	02	01	06	0	15
37–48 months	02	01	01	00	04	01	10
Total	31					32	60

In our study, among the partially vaccinated cases, all patients took 3 doses of vaccination or less. The recommended number of doses of Oral Polio Vaccine (OPV) is between 9–12 for children to reach sufficient immunity. The children in the control group received all the doses during the campaign, therefore showing no disease symptoms.

To assess the knowledge about polio we conducted a survey-based study from March to September 2019. Out of 500 people approached, 384 individuals agreed to participate in the study. Table 3 shows how participants were categorised based on their demographic data like age, gender, education, marital status and employment status, etc.

**Table 3:** Association of demographic variables with the knowledge of people towards Poliomyelitis.

Variables		total no.	Knowledge (%)		OR (95%CI)	p
			Poor	Good		
Age (years)						
	18–30	149	52 (34.9 %)	97 (65.1 %)	0.9911 (0.9032–1.087)	0.8486
	31–40	99	62 (62.6 %)	37 (37.4 %)	0.9955 (0.8592–1.154)	0.9518
	41–50	65	31 (47.7 %)	34 (52.3 %)	0.9127 (0.7722–1.073)	0.272
	51–60	41	34 (82.9 %)	07 (17.1 %)	0.8775 (0.6306–1.173)	0.3949
	> 60	30	27 (90.0 %)	03 (10.0 %)	0.9612 (0.7182–1.176)	0.733
Gender						
	Female	225	128 (56.9 %)	97 (43.1 %)	1.019 (0.9289–1.117)	0.6945
	Male	159	90 (56.6 %)	69 (43.4 %)	0.9787 (0.8772–1.091)	0.6981
Qualification						
Uneducated		72	53 (73.6 %)	19 (26.4 %)	1.031 (0.8600–1.239)	0.7401
	Primary	58	38 (65.5 %)	20 (34.5 %)	1.099 (0.9063–1.344)	0.3381
	Secondary	106	64 (60.4 %)	42 (39.6 %)	0.987 (0.8612–1.131)	0.8542
	Religious	87	53 (60.9 %)	34 (39.1 %)	1.006 (0.8646–1.171)	0.9352
Graduate		61	33 (54.1 %)	28 (45.9 %)	0.9099 (0.7589–1.083)	0.2939
Employment status						
	Unemployed	168	113 (67.3 %)	55 (32.7 %)	1.027 (0.9173–1.150)	0.6463
	Paid-employed	115	67 (58.3 %)	48 (41.7 %)	0.9634 (0.8432–1.099)	0.5794
	Self-employed	101	63 (62.4 %)	38 (37.6 %)	0.9693 (0.8403–1.116)	0.665
Monthly Income (PKR)						
	< 10,000–20,000	168	113 (67.3 %)	55 (32.7 %)	1.034 (0.9248–1.157)	0.5559
	< 30,000–50,000	115	67 (58.3 %)	48 (41.7 %)	1.006 (0.8846–1.145)	0.9233
	> 50,000	101	51 (50.5 %)	50 (49.5 %)	1.013 (0.8843–1.161)	0.8502
Marital status						
	Single	41	16 (39.0 %)	25 (61.0 %)	1.134 (0.9126–1.431)	0.2662
	Married	343	96 (28.0 %)	246 (72.0 %)	0.9984 (0.9197–1.084)	0.9688
Participants having children < 5 years of age						
	No	171	104 (60.8 %)	67 (39.2 %)	1.005 (0.9027–1.119)	0.9276
	Yes	215	116 (54.0 %)	99 (46.0 %)	1.003 (0.9126–1.102)	0.9564
Past experience with polio patients						
	No	243	167 (68.8 %)	76 (31.2 %)	0.9827 (0.8938–1.080)	0.7159
	Yes	141	52 (36.9 %)	89 (63.1 %)	1.006 (0.8923–1.134)	0.9278
Residence						
Peshawar outskirts		291	189 (65.0 %)	102 (35.0 %)	0.9998 (0.9192–1.087)	0.9965
Peshawar city		93	46 (49.5 %)	47 (50.5 %)	0.813 (0.6948–0.9412)	0.0071

Participants’ knowledge was assessed based on a questionnaire specially designed for this study. The questionnaire was comprised of 15 questions related to poliomyelitis and its effects. Most of the people (77.3%) were familiar with the name of the disease “polio”. About 62.5% of people were aware of the cause of the disease. There was little difference between the individuals who knew about polio's symptoms and the people who were unaware. Roughly 75.3% of people were aware of the treatment of polio. About 51.6% of people agreed that there were some side effects of the polio vaccine. Around 75.5% of participants denied that polio is transmittable. By assessing participants’ knowledge, we observed that people were aware of the polio disease due to the polio campaign, but most of the people were unaware of the clinical symptoms and post-polio syndrome. Furthermore, participants had false religious beliefs and misconceptions about the vaccine (like it could cause HIV or sterility in children), which is why they were reluctant to immunise their children. The mean knowledge score of the participants was 7.64.

The assessment revealed that individuals between the age of 18–30 years had good knowledge (65.1%) about polio compared to the senior individuals (10.0%) who had poor knowledge about the disease (Table 3). Male and female participants were equally aware about the disease (~ 43%). We observed a significant impact of education on people's opinion about general health. Participants with higher education had more knowledge (45.9%) than people without any formal education (34.5%). Interestingly, individuals with better economic positions had more awareness (49.5%) than low-income participants (32.7%). Participants having children less than 5 years of age had more knowledge (46%). Individuals who had a previous experience with a polio patient in their family or neighbours were more aware of the disease (63.1%). Finally, participants living in the city were better aware (50.5%) of polio than the individuals living in Peshawar city outskirts (35%).

**Figure 2:**
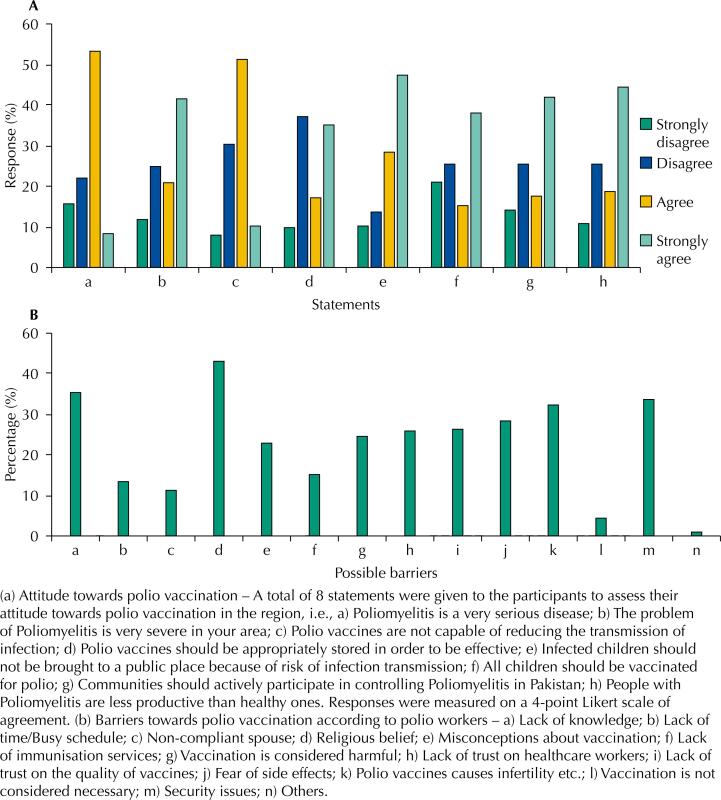
Assessment of participants attitude towards polio vaccination (a) and possible barriers towards polio vaccination according to polio workers (b).

Figure 2a shows that 15.8% of participants were still not considering polio as a severe disease. About 21% of individuals strongly disagreed that children should be vaccinated and 14.5% of individuals strongly disagreed on community participation for the polio eradication from the country. Around 10.9% of participants strongly rejected the statement that polio patients are less productive than those physically normal and healthy.

During our study, we also tried to identify the possible barriers in the way of polio immunisation. For this purpose, 178 polio workers were approached and interviewed during a polio campaign in April 2019. Many issues were identified on those interviews. Figure 2 (b) shows that religious disbelief (43.2%) – i.e., the polio vaccine had some content that is unlawful to consume according to some uneducated religious leaders – is the biggest hurdle to immunization. Lack of knowledge is the second biggest problem, 35.4 %, in the way of polio eradication from Pakistan according to the respondents^18^.

We also tried to get people's perceived concern about polio vaccination. Most of them had believed that the polio vaccine has some contents which are not halal to consume, and this is true to some extent. The polio vaccine contains a small amount of trypsin, which is sometimes extracted from pork (which is forbidden in Islam). People also showed concern towards the quality of polio vaccine. Many participants had a false belief that there is some hidden agenda behind the Polio eradication program to target religious communities or coloured races.

## DISCUSSION

Pakistan is reported as the only country with consistent barriers preventing vaccination. The Government of Pakistan has handled the problems by involving social mobilisers and religious leaders^19^. However, the recent re-emergence of polio cases, especially in KPK, is very concerning at national and international levels. Controlling the disease will need additional strict steps over the years ahead^20^.

Figure 1 shows data from a province-wide poliomyelitis report revealing that the number of polio cases was highest in the region of KPK. Our study highlighted the factors causing the rise of poliomyelitis by assessing the local community's perception and attitude towards the immunisation scheme, specifically in Peshawar. The study assessed the opinions of both the general public and the vaccination team by questionnaire.

In general, this study concluded a lack of knowledge about viral transmission and inappropriate dosage are the main causes. The children getting insufficient doses (only three doses) were affected by polio (Table 2). Uthman et al.^21^ (2017) observed similar cases in Nigeria where illiterate mothers’ refusal to immunise their children, or incomplete course of recommended vaccine doses, was found to cause polio transmission among children as described in other published studies^22^. With the government social mobilization, people are getting familiarised with the disease name and cause, but the knowledge about transmission and dosage of vaccine needs to be conveyed. Our study proved a significant change in people's preliminary knowledge as compared with two similar studies conducted by Khan et al. in 2015^2^ and Habib et al. in 2017^23^.

Our study revealed that most of the individuals agreed that polio is a severe disease. Still, they were reluctant to get their children immunized due to the lack of awareness about the possible threats. We found the older people had poorer knowledge than the youngsters (below 30) since they were less familiar with social media and were mostly illiterate. Thus, ignorance became a significant factor that influenced the vaccination rate in Pakistan, as reported by Habib et al.^23^, Khan et al.^2^, Hussain et al.^24^, and Lorenz and Khalid^25^. Campaigns or seminars to educate people in communities should involve trustworthy personnel like area counsellors or tehsildar to convey detailed information in their local language to modify people's perception of the disease. Despite the Government of Pakistan's relentless efforts, the polio cases are still increasing and people are not fully convinced to vaccinate their children, as reported by Waheed et al.^26^ in 2018.

In our study, the polio workers/volunteers were also interviewed as they are the frontline workers and those coping with the community resistance towards polio campaigns (Figure 3). Most people in Pakistan blindly believe preachers’ opinions about specific products; therefore, altering their mindset is difficult. Similar issues were reported by Khan et al.^27^ in 2017, and people still have a negative attitude towards the vaccine.

We found that most people had false beliefs regarding the polio vaccine; for example, the vaccine is below the international standard or is stored incorrectly. Some even believe it to be harmful or cause infertility or HIV infection.

During the study, people usually get uncomfortable because of frequent visits of the male polio workers and mothers at home avoid the interaction. Considering this issue, the government of Pakistan specifically trained female health workers to satisfy the parents. We observed that some criminals took advantage of this campaign to loot, making people distrust the polio teams. The polio team worker background should be checked before field visits.

This study found that some parents believed vaccines could make their children sick. The belief was strengthened after an incident in Peshawar when on the second day of the polio vaccination campaign, hundreds of children rushed to hospitals complaining of abdominal pain, diarrhea, and vomiting. Consequently, the parents in rural and urban areas of Peshawar and the capital of Islamabad refused their children to be vaccinated the next day, on April 2017^28^. The communication officers and mobilization teams struggle to convince people that repeated polio vaccinations are safe, and that complete immunization is needed for children's immune system to cope with the virus, yet this message is still not effectively communicated^17^. Polio workers also noted that political and religious leaders’ negative attitudes prompt security issues, which might interfere with their performance. For example, a policeman was gunned down while protecting polio workers during a polio campaign in Bannu region of KPK in 2019^29^. Similar problems were reported in a study conducted by Bham et al^30^.

To eradicate polio from Pakistan, we must overcome the religious, political and socio-economic obstacles to immunisation, including vaccination coverage gaps, inadequate health facilities and tensions in the country's polio-endemic areas^22^.

The scholars or religious leaders should be sensitized to the severity of the disease and its control to clear their insecurities. In that way, we could change the mindset of a vast mass.

### Limitations in Study

Despite the many efforts to make this study possible, it has a few limitations. First, the study area was confined to a single city and a comparison with other cities of the province should be made to get a clear picture.

Secondly, the resistance of people difficulted the data collection. People in this locality usually do not want to take part in such studies, especially when they are doubtful due to false beliefs. Lastly, the study was survey-based without any experimental work. A study could be designed to measure the magnitude of the disease spread by testing water samples from sewage and other possible sources.

## CONCLUSION

By analysing the data gathered in this study, we concluded that despite the government's extensive polio eradication program, Pakistan is still behind the rest of the world in the battle against poliomyelitis. The factors behind the polio campaign's failure include low literacy rate, insufficient knowledge, false religious beliefs, unchecked migration between Afghanistan and Pakistan, lack of health facilities, lack of trust in health workers or vaccines, and security threats to the polio workers in the region.
